# MicroRNA and gene networks in human diffuse large B-cell lymphoma

**DOI:** 10.3892/ol.2014.2438

**Published:** 2014-08-12

**Authors:** KUNHAO WANG, ZHIWEN XU, NING WANG, TING XU, MINGHUI ZHU

**Affiliations:** 1 Department of Computer Science and Technology, Jilin University, Changchun, Jilin 130012, P.R. China; 2 Key Laboratory of Symbolic Computation and Knowledge Engineering of Ministry of Education, Jilin University, Changchun, Jilin 130012, P.R. China; 3 Department of Economics, Changchun University, Changchun, Jilin 130022, P.R. China

**Keywords:** DLBCL, miRNA, TFs, network, host gene

## Abstract

Molecular biologists have collected considerable data regarding the involvement of genes and microRNAs (miRNAs) in cancer. However the underlying mechanisms of cancer with regard to genes and miRNAs remain unclear. The aim of the present study was to evaluate diffuse large B-cell lymphoma (DLBCL) and construct regulatory networks of genes and miRNAs to gradually reveal the underlying mechanisms of DLBCL development. The first differential expression network that is presented is an experimentally validated network of miRNAs and genes. This network presents known biological regulatory associations among miRNAs and genes in the human body. The second network is a DLBCL differential expression network. Differentially expressed gene and miRNA data regarding DLBCL were collected and, based on the first network and the differentially expressed data, the second network was inferred, which demonstrates the irregular regulatory associations that may lead to the occurrence of DLBCL. The third network is a DLBCL-associated network. This network is comprised of non-differentially expressed genes and miRNAs that contribute to numerous DLBCL processes. The similarities and differences among the three networks were extracted and compared to distinguish key regulatory associations; furthermore, important signaling pathways in DLBCL were identified. The present study partially clarified the pathogenesis of DLBCL and provided an improved understanding of the underlying molecular mechanisms, as well as a potential treatment for DLBCL.

## Introduction

Diffuse large B-cell lymphoma (DLBCL) is the most common subtype of non-Hodgkin lymphoma (NHL) and constitutes a heterogeneous category of aggressive lymphomas (1). Numerous studies have shown that genes and microRNAs (miRNAs) exert various roles in DLBCL (2–5). Furthermore, differentially expressed genes and miRNAs are important in the pathogenesis of DLBCL; for example, *TRX1* is key in cell growth and survival, as well as in the chemoresistance of relapsed/refractory DLBCL (6). In addition, the misregulation of hsa-miR-155 and hsa-miR-146a acts as a diagnostic and prognostic marker (7). Genes and miRNAs that are not differentially expressed, but are associated with DLBCL also exert particular roles in DLBCL. The B cell receptor isotype is a reliable indicator for the *GCB* and *ABC* subtypes in DLBCL (8), and hsa-miR-135b contributes to tumorigenesis through modulation of the tumor immune-phenotype and microenvironment (9).

Gene regulatory factors are predominantly comprised of transcription factors (TFs) and miRNAs, and these control the expression of genomic information in multicellular genomes (10). TFs are proteins that bind to specific DNA sequences, controlling the transfer of genetic information between DNA and messenger RNA (11). TFs may regulate (activate or repress) gene expression alone or in conjunction with other proteins. miRNAs are small non-coding RNA molecules (~22 nucleotides in length) that function in the transcriptional and post-transcriptional regulation of gene expression (12). miRNAs regulate gene expression by silencing genes or targeting genes for degradation, and influence various cancer processes, including proliferation, differentiation and apoptosis.

miRNAs target thousands of human genes, usually known as target genes (targets), which are important in analyzing the biological functions of miRNAs. Currently, a number of arithmetic methods (13) and experimentally validated databases (14,15) have provided sufficient data to investigate the associations among different miRNAs.

In DNA sequences, miRNAs are encoded by certain genes; these are usually termed miRNA host genes. In the transcription process, miRNAs and the corresponding host gene are transcribed simultaneously (16). The host gene and the intronic miRNA are coordinately expressed in certain biological processes (17), and together accomplish certain functions and are involved in signaling pathways (18).

The occurrence of DLBCL cannot be attributed to a single gene, miRNA or signaling pathway; rather, DLBCL is the result of various biological functions acting together. A number of genes and miRNAs associated with DLBCL have been identified; however, the underlying mechanism of miRNA and gene involvement in DLBCL remains largely unknown. The present study focused on the networks of TFs, miRNAs, miRNA targets and miRNA host genes to examine the key regulatory associations in DLBCL and partially reveal the underlying control mechanisms. Experimentally validated associations (among TFs and miRNAs, miRNAs and the respective target genes, and miRNAs and the corresponding host genes) were collected from TarBase, miRTarBase, TransmiR and miRBase databases (14,15,19,20). Differentially expressed genes and miRNAs in DLBCL, and DLBCL-associated genes and miRNAs were collected from databases and the relevant literature. To further investigate the DLBCL transcriptional network, TFs were obtained by the P-match method and were considered as DLBCL-associated genes. The associated genes and miRNAs included those that were differentially and non-differentially expressed. Three networks were constructed to gradually understand the mechanism of DLBCL. The first is an experimentally validated network of miRNAs and genes constructed from all data. The second is a differential expression network; differentially expressed genes and miRNAs were mapped onto the first network, then extracted to construct the second network. The third is a DLBCL-associated network (similar methods were used to construct this third network). The regulatory associations between differentially expressed genes, differentially expressed miRNAs and predicted TFs were separately extracted from the three networks, and the similarities and differences were compared to distinguish the key regulatory associations in DLBCL.

## Materials and methods

### Data collection and processing

An experimentally validated dataset of miRNAs and the corresponding targets were extracted from TarBase 5.0 (Diana Lab, Philadelphia, PA, USA) (14) and miRTarBase (15). The National Center for Biotechnology Information (NCBI) gene database (http://www.ncbi.nlm.nih.gov/gene/) was used to unify the official symbols of miRNAs and genes; this dataset was designated set *U*_1_.

An experimentally validated dataset of TFs and miRNAs was extracted from the TransmiR database (http://www.cuilab.cn/transmir) (19), and this dataset was termed set *U*_2_.

The dataset of host genes and the respective miRNAs was extracted from the miRBase (http://www.mirbase.org/)(20) and NCBI gene databases. This dataset was set *U*_3_.

In the present study, the differentially expressed genes included genetically mutated genes, abnormally expressed protein genes, single nucleotide polymorphisms (SNPs) and overexpressed, downregulated, upregulated genes. The dataset of differentially expressed genes was retrieved from Cancer Genetics Web (http://www.cancerindex.org/geneweb/index.html) and the relevant literature was obtained from the NCBI SNP database (http://www.ncbi.nlm.nih.gov/snp/).

Numerous DLBCL-associated genes have been detected, such as those involved in the development and metastasis of human DLBCL, as well as those used therapeutically in DLBCL prevention, diagnosis and radial therapy. The DLBCL-associated genes also include the differentially expressed genes. The dataset of DLBCL-associated genes was collected from the GeneCards database(http://www.genecards.org/) (21) and the relevant studies found using PubMed. To gain an improved understanding of the transcriptional network of TFs, miRNAs and targets, popular TFs were extracted using the P-match method (Biobase, Wolfenbüttel, Germany) (22). These TFs are termed the DLBCL-associated genes. The present study focused on the TFs that appear on the TransmiR database. Since miRNAs regulate gene expression together with TFs, 1,000-nt promoter region sequences of targets that are targeted by differentially expressed miRNAs were downloaded from the University of California, Santa Cruz Genome Browser (23). The P-match method was used to identify TF binding sites (TFBSs) in the 1,000-nt promoter region sequences. These TFBSs were mapped onto the promoter regions of targets, then the corresponding TFs of these TFBSs were obtained. In the P-match method, the vertebrate matrix was selected with a high-quality criterion for the extracted TFs. The dataset of differentially expressed and associated genes was set *U*_4_.

Differentially expressed miRNAs include overexpressed, downregulated and upregulated miRNAs. The associated miRNAs involved in various DLBCL processes include differentially expressed and non-differentially expressed miRNAs. The dataset of differentially expressed miRNAs was retrieved from the mir2Disease database (http://www.miR2Disease.org (24). The dataset of DLBCL-associated miRNAs was collected from the differentially expressed miRNA data and information from previous relevant studies; this dataset was termed set *U*_5_.

### Construction of the three networks

To construct the experimentally validated, differentially expressed and associated networks, regulatory associations among TFs, miRNAs, targets and host gene were extracted from *U*_1_, *U*_2_ and *U*_3_, and the associations were combined to construct the experimentally validated network. Differentially expressed genes and miRNAs were extracted from *U*_4_ and *U*_5_, then mapped onto the experimentally validated network. The differential expression network was constructed by extracting these associations and combining them. Certain differentially expressed genes and miRNAs were not present in *U*_1_, *U*_2_ and *U*_3_; these were presented as single nodes in the differential expression network. Similar methods were used to construct the DLBCL-associated network.

## Results

### DLBCL differential expression network

**Figure 1:**
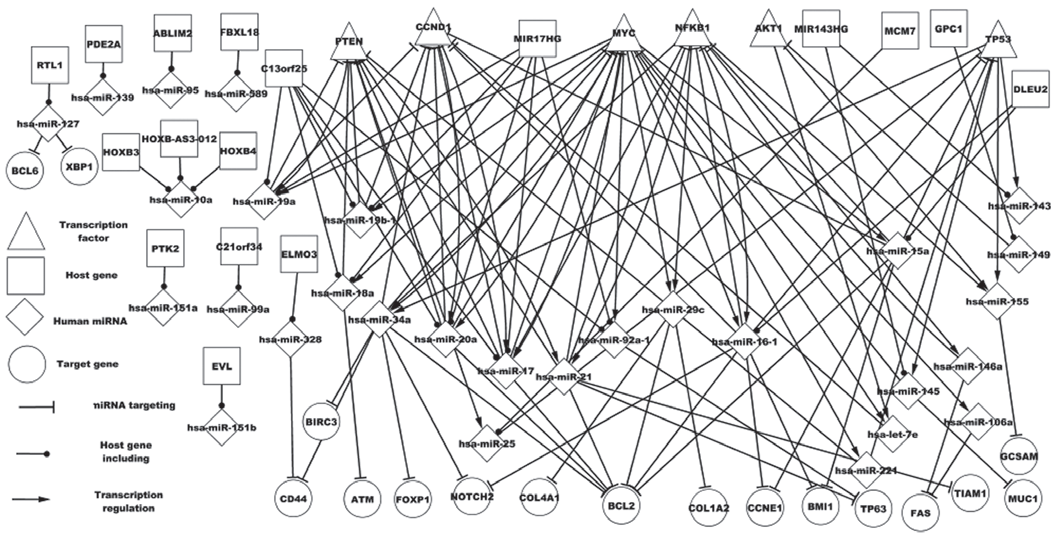
Differential expression network of genes and miRNAs in DLBCL. With the exception of certain host genes, all nodes are involved in various DLBCL processes, including the cell cycle (TP53, CCND1, hsa-miR-145 and hsa-miR-21), tumor metastasis (BMI1) and tumor growth (hsa-miR-145 and NFKB1).miRNA, microRNAs; DLBCL, diffuse large B-cell lymphoma.

A number of important miRNAs, genes and the corresponding regulatory associations may result in the occurrence of DLBCL (Fig. 1). All single nodes that do not have a regulatory association with miRNAs, such as BCL10 and CASP10, were omitted, although these nodes also exert key roles in DLBCL. A total of six TFs, 23 miRNA targets, and 21 miRNAs and the respective host genes are presented in Fig. 1. With the exception of certain host genes, the other nodes all indicate differentially expressed miRNAs and proteins in DLBCL. Fig. 1 demonstrates various types of regulatory association between miRNAs and genes. One miRNA may target one gene or numerous genes, one TF may regulate one miRNA or numerous miRNAs, numerous TFs may regulate one miRNA or numerous miRNAs, and numerous miRNAs may target one gene or numerous genes. Particular features of host genes and the respective miRNAs are revealed in Fig. 1. A host gene may encode one or numerous miRNAs that target other genes; for example GPC1 encodes hsa-miR-149, which targets AKT1. An miRNA may be located in various host genes; for example, three host genes (HOXB3, HOXB4 and HOXB-AS3-012) encode hsa-miR-10a. Thus, the differential expression network partially revealed the regulatory mechanism of DLBCL.

### DLBCL-associated network

Numerous regulatory associations among genes and miRNAs were identified in the DLBCL-associated network. Compared with the differential expression network, the DLBCL-associated network includes additional TFs, miRNAs and mass targets. The DLBCL-associated network also reveals further regulatory associations between genes and miRNAs; for example, *EGFR* was found to regulate hsa-miR-21, which targets MYC; hsa-miR-21 was identified to target E2F1, which regulates hsa-miR-17; and TP53 was revealed to regulate hsa-miR-125b (hsa-miR-125b-1 and -2), which targets AKT1. These nodes are all associated with DLBCL, however, certain nodes do not demonstrate differentially expressed data. The DLBCL-associated network expands on the differential expression network and these newly identified regulatory associations may contribute to tumor growth, migration, prevention, diagnosis, development and other processes in DLBCL.

### Host genes and the corresponding miRNAs in DLBCL

Two differentially expressed genes (TP63 and FOXP1) were identified as host genes in the present study, although the respective miRNAs were not differentially expressed in DLBCL. hsa-miR-127 was found to be encoded in RTL1, and to target two differentially expressed genes, XBP1 and BCL6. Although certain host genes are not differentially expressed in DLBCL, the genes may be involved in certain DLBCL processes when the miRNAs are differentially expressed. In the differential expression network, certain host genes and the corresponding miRNAs exhibit the feature where a host gene encodes numerous miRNAs that alone or together target specific genes.

**Figure 2:**
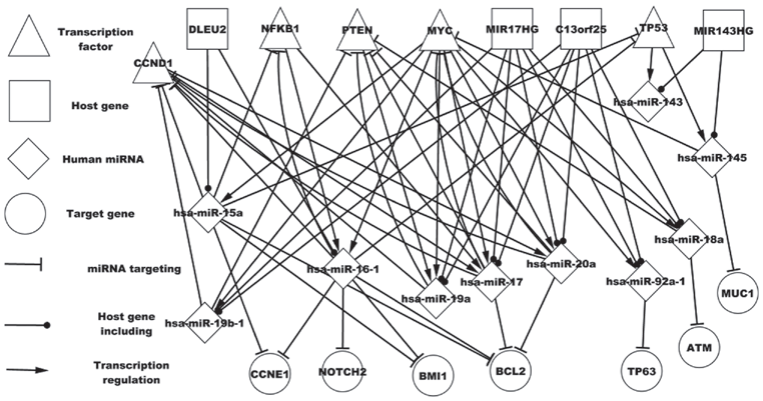
Important associations between host genes and miRNAs in the diffuse large B-cell lymphoma differential expression network. A host gene may encode various miRNAs that target certain genes either alone or together. Particular miRNAs may be encoded by one host gene, which is regulated by a transcription factor. miRNA, microRNA.

Fig. 2 shows certain particular host genes and the regulatory associations between these genes and TFs, and miRNAs and the corresponding targets. For example, MIR17HG encodes six miRNAs, and four of these miRNAs (hsa-miR-17, hsa-miR-20a, hsa-miR-19a and hsa-miR-19b-1) target CCND1. hsa-miR-19a is regulated by MYC and PTEN and hsa-miR-19a and PTEN form a self-adaptation association. hsa-miR-17 is regulated by three TFs (MYC, NFKB1 and CCND1) and MIR143HG encodes two miRNAs (hsa-miR-143 and hsa-miR-145) that are regulated by TP53. hsa-miR-145 targets MUC1, however, hsa-miR-143 does not target any differentially expressed genes. hsa-miR-17 and hsa-miR-16-1 are regulated by NFKB1, which is targeted by hsa-miR-15a and hsa-miR-16-1. In conclusion, host genes and the respective miRNAs may aid in understanding the pathogenesis of DLBCL.

### Transcriptional network of predicted TFs

**Figure 3:**
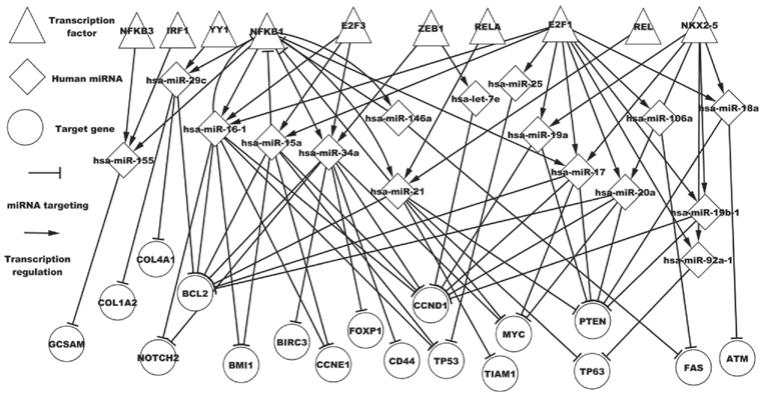
Transcription network of predicted TFs, and differentially expressed miRNAs and the respective target genes in diffuse large B-cell lymphoma. These predicted TFs are frequently involved in cancer transcription processes. TFs, transcription factors; miRNA, microRNA.

A total of 16 differentially expressed miRNAs regulated by the predicted TFs were further analyzed. Fig. 3 shows the regulatory associations among predicted TFs, differentially expressed miRNAs and differentially expressed targets in DLBCL. These TFs and miRNAs in turn influence the respective successors. For example, the E2F1, PAX5, REL, RELA, STAT1 and YY1 TFs have been experimentally validated in DLBCL. NFKB1 is a differentially expressed TF gene in DLBCL. Fig. 3 shows that NFKB1 regulates seven miRNAs and is targeted by four miRNAs. hsa-miR-146a, hsa-miR-21, hsa-miR-16-1 and NFKB1 form three self-adaptation associations. NFKB1 regulates hsa-miR-17, which targets MYC, CCND1, PTEN and BCL2. Fig. 3 also shows that a differentially expressed miRNA may be regulated by various TFs, a target may be targeted by numerous differentially expressed miRNAs, an miRNA may indirectly influence other miRNAs through particular TFs and a TF may indirectly influence other genes through various differentially expressed miRNAs. For example, hsa-miR-29c is regulated by YY1 and NFKB1; hsa-miR-17, hsa-miR-21, hsa-miR-20a and hsa-miR-34a target MYC: YY1 regulates hsa-miR-29c, which targets BCL2; and hsa-miR-21 targets NFKB1, which regulates hsa-miR-155. This transcription network may contribute to the further understanding of DLBCL pathogenesis.

### Regulatory associations among differentially expressed genes

To understand the regulatory network more clearly, the regulatory associations of each node (differentially expressed genes, differentially expressed miRNAs and predicted TFs) were extracted and compared according to the predecessors and successors of the gene, the nodes preceeding the current one on the path or the node following the current one on the path, respectively. Among these genes, MYB, MYC and CCND1 and four miRNAs (hsa-miR-17, hsa-miR-155, hsa-miR-15a and hsa-miR-34a) formed five self-adaptation associations.

For the differentially expressed genes, PTEN may be used as an example. Table I shows PTEN, and the respective predecessors and successors in the three networks. Seven miRNAs target PTEN-mediated regulation of three miRNAs in the differential expression network, eight miRNAs target PTEN-mediated regulation of three miRNAs in the DLBCL-associated network and 21 miRNAs target PTEN-mediated regulation of nine miRNAs in the experimentally validated network. In the human body, the regulatory associations between PTEN and DLBCL-associated miRNAs influence multiple DLBCL processes. However, the regulatory associations between PTEN and the non-associated miRNAs may not influence DLBCL. Predecessors may indirectly influence successors by regulation of PTEN. Two miRNAs (hsa-miR-19a and hsa-miR-21) were found to target PTEN (Table I) and form two self-adaptation associations. Furthermore, hsa-miR-19a and hsa-miR-21 are also regulated by PTEN. The expression of another miRNA may subsequently be affected when either of these miRNAs are differentially expressed.

### Regulatory associations among differentially expressed miRNAs

As with the differentially expressed genes, the regulatory associations among differentially expressed miRNAs were extracted and compared according to the predecessors and successors of miRNA.

The analysis only focused on hsa-miR-20a to illustrate the regulatory associations regarding differentially expressed miRNAs in three networks (differentially expressed, related and experimentally validated networks). Table II shows hsa-miR-20a, and its predecessors and successors in the three networks. CCND1 and MYC regulate hsa-miR-20a, which targets four genes in the differential expression network. In the DLBCL-associated network, four genes regulate hsa-miR-20a, which targets 14 genes. In the experimentally validated network, 10 genes regulate hsa-miR-20a, which targets 26 genes. MYC, CCND1 and hsa-miR-20a were found to form two self-adaptation associations (Table II). As with PTEN, regulatory associations between hsa-miR-20a and DLBCL-associated genes influence multiple DLBCL processes; however, other regulatory associations between hsa-miR-20a and non-associated genes may not influence DLBCL.

### Regulatory associations among predicted TFs

The predecessors and successors of the predicted TFs were used to extract and compare the regulatory associations of each predicted TF. Three TFs (E2F1, E2F3 and NFKB1) and five differentially expressed miRNAs form five self-adaptation associations. Notably, NFKB1 is a differentially expressed gene and E2F1 is associated with DLBCL.

The present study only focused on E2F3 to illustrate regulatory associations regarding differentially expressed miRNAs in three networks (differentially expressed, related and experimentally validated networks). Table III shows E2F3, and its predecessors and successors in the three networks. Four differentially expressed miRNAs target E2F3, which regulates three differentially expressed miRNAs. In the DLBCL-associated network, six miRNAs target E2F3, which regulates three miRNAs in the same network. In the experimentally validated network, 12 miRNAs target E2F3, which regulates 11 miRNAs. hsa-miR-34a and E2F3 were found to form a self-adaptation association in the differential expression network. In this self-adaptation association, E2F3 is not differentially expressed in DLBCL, although hsa-miR-34a is differentially expressed. Therefore, hsa-miR-34a may indirectly cause other miRNAs to be erroneously expressed by E2F3.

## Discussion

Certain important regulatory associations among differentially expressed genes, differentially expressed miRNAs and predicted TFs were identified in the present study. For the differentially expressed genes and miRNAs, the results indicated numerous important regulatory associations, including the fact that hsa-miR-149 targets AKT1, which regulates hsa-let-7e. The signaling pathways identified may exert key biological functions in DLBCL or affect normal physiological processes, thus resulting in the occurrence of DLBCL. Certain signaling pathways have been detected that influence particular processes in DLBCL; for example hsa-miR-155 regulates the PI3K-AKT signaling pathway in DLBCL (25). Other signaling pathways have not been identified in DLBCL, however, may influence numerous processes in other types of cancer; for example, hsa-miR-15a/hsa-miR-16-1 targets BCL2 and exerts an etiological and therapeutic role in keratocystic odontogenic tumors (26), and hsa-miR-16-1 targets CCND1 in mantle cell lymphoma (27). Poliseno *et al* (28) observed that PTEN regulates hsa-miR-25 and Kumar *et al* (29) indicated that TP53 is targeted by hsa-miR-25. hsa-miR-25 is differentially expressed in DLBCL, and forms an association between PTEN and TP53; PTEN may influence TP53 expression via hsa-miR-25. The present study may expand the understanding of the associations among these genes. Although certain signaling pathways were not found to influence DLBCL processes in the present study, the biological functions of these signaling pathways in other types of cancer may contribute to DLBCL. The remaining signaling pathways, which have not been associated with any type of cancer, may exert potential functions in DLBCL; for example, in the present study, hsa-miR-92a-1 was found to target TP63. For the predicted TF signaling pathways, certain signaling pathways have been detected in other types of cancer; for instance, ZEB1 regulates hsa-miR-34a in lung cancer (30) and hsa-miR-21 targets E2F2 in breast cancer (31).

In conclusion, in the present study, numerous important regulatory associations in DLBCL were identified. Furthermore, certain genes and miRNAs exhibited self-adaptation associations. The differential expression network partially revealed the pathogenesis of DLBCL and the DLBCL-associated network supplied comprehensive data with regard to the genes and miRNAs associated with DLBCL processes, including prevention, diagnosis, development and therapy. Therefore, the present study contributes to the understanding of the underlying molecular mechanisms and potential treatment of DLBCL.

## Figures and Tables

**Table I:** Regulatory associations between miRNAs and PTEN.

miRNAs that target PTEN			miRNAs regulated by PTEN		
	
Differential expression network	Associated network	Experimentally validated network	Differential expression network	Associated network	Experimentally validated network
		miR-106b			
		miR-141			
		miR-17			
		miR-18a			
		miR-19a			
		miR-19b-1			
miR-17	miR-17	miR-19b-2	miR-19a	miR-19a	miR-19a
miR-18a	miR-18a	miR-20a	miR-21	miR-21	miR-21
miR-19a	miR-19a	miR-21	miR-25	miR-25	miR-22
miR-19b-1	miR-19b-1	miR-214			miR-25
miR-20a	miR-20a	miR-216a			miR-302a
miR-21	miR-21	miR-221			miR-302b
miR-221	miR-221	miR-222			miR-302c
	miR-222	miR-26a-1			miR-302d
		miR-26a-2			miR-302f
		miR-29b			
		miR-217			
		miR-494			
		miR-519a			
		miR-519d			
		miR-93			

miRNA, microRNA.

**Table II:** Regulatory associations between hsa-miR-20a and various genes.

Genes that regulate hsa-miR-20a			Genes targeted by hsa-miR-20a		
	
Differential expression network	Associated network	Experimentally validated network	Differential expression network	Associated network	Experimentally validated network
CCND1	CCND1	CCND1	BCL2	CCND1	APP, CCND1
MYC	E2F1	E2F1	CCND1	BCL2	BCL2, BMPR2
	MYC	MYC	MYC	RUNX1	BNIP2, RUNX1
	NKX2-5	MYCN	PTEN	CCND2	CCND2, CDKN1A
		NKX2-5		CDKN1A	E2F1, E2F3
		TLX1		E2F1	HIF1A, IRF2
		TLX3		E2F3	KIT, SMAD4
		ESR1		HIF1A	MEF2D, MYC
		STAT5B		IRF2	NRAS, MAPK9
		SPI1		KIT	PTEN, RB1
				MYC	RBL1, RBL2
				PTEN	TGFBR2, THBS1
				RB1	VEGFA, WEE1
				TGFBR2	MAP3K12, EGLN3
				THBS1	MUC17
				VEGFA	

miRNA, microRNA.

**Table III:** Regulatory associations between miRNAs and the transcription factor, E2F3.

miRNAs that target E2F3			miRNAs regulated by E2F3		
	
Differential expression network	Associated network	Experimentally validated network	Differential expression network	Associated network	Experimentally validated network
miR-17	miR-125b-1	miR-106b	miR-15a	miR-15a	let-7a-1
miR-20a	miR-125b-2	miR-125b-1	miR-16-1	miR-16-1	let-7a-2
miR-210	miR-17	miR-125b-2	miR-34a	miR-34a	let-7a-3
miR-34a	miR-20a	miR-128b			miR-15b
	miR-210	miR-17			miR-15a
	miR-34a	miR-195			miR-16-1
		miR-20a			miR-16-2
		miR-200b			miR-195
		miR-203a			miR-106b
		miR-210			miR-34a
		miR-34a			let-7i
		miR-34c			

miRNA, microRNA.

## Abbreviations

miRNA: microRNA
TFs: transcription factors
targets: target genes
DLBCL: diffuse large B-cell lymphoma
NCBI: National Center for Biotechnology Information
TFBSs: transcription factor binding sites
NHL: non-Hodgkin lymphoma
